# Regular mosaic pattern development: A study of the interplay between lateral inhibition, apoptosis and differential adhesion

**DOI:** 10.1186/1742-4682-4-43

**Published:** 2007-10-31

**Authors:** Gregory J Podgorski, Mayank Bansal, Nicholas S Flann

**Affiliations:** 1 Biology Department and Center for Integrated Biosystems, Utah State University, Logan UT, USA; 2 Computer Science Department, Utah State University, Logan UT, USA

## Abstract

**Background:**

A significant body of literature is devoted to modeling developmental mechanisms that create patterns within groups of initially equivalent embryonic cells. Although it is clear that these mechanisms do not function in isolation, the timing of and interactions between these mechanisms during embryogenesis is not well known. In this work, a computational approach was taken to understand how lateral inhibition, differential adhesion and programmed cell death can interact to create a mosaic pattern of biologically realistic primary and secondary cells, such as that formed by sensory (primary) and supporting (secondary) cells of the developing chick inner ear epithelium.

**Results:**

Four different models that interlaced cellular patterning mechanisms in a variety of ways were examined and their output compared to the mosaic of sensory and supporting cells that develops in the chick inner ear sensory epithelium. The results show that: 1) no single patterning mechanism can create a 2-dimensional mosaic pattern of the regularity seen in the chick inner ear; 2) cell death was essential to generate the most regular mosaics, even through extensive cell death has not been reported for the developing basilar papilla; 3) a model that includes an iterative loop of lateral inhibition, programmed cell death and cell rearrangements driven by differential adhesion created mosaics of primary and secondary cells that are more regular than the basilar papilla; 4) this same model was much more robust to changes in homo- and heterotypic cell-cell adhesive differences than models that considered either fewer patterning mechanisms or single rather than iterative use of each mechanism.

**Conclusion:**

Patterning the embryo requires collaboration between multiple mechanisms that operate iteratively. Interlacing these mechanisms into feedback loops not only refines the output patterns, but also increases the robustness of patterning to varying initial cell states.

## Background

Pattern formation is a defining feature of biological development. Many mechanisms account for the emergence of complex patterns within a group of initially equivalent cells, including lateral inhibition, differential adhesion, programmed cell death, cell migration, differential growth, and asymmetric cell division [1]. A rich literature describes computational models of each of these patterning processes and explores how these mechanisms can generate the patterns observed during development [2,3]. These modeling studies have offered invaluable insights. However, the vast majority of earlier computational models have explored the role of individual patterning mechanisms, whereas within the embryo these mechanisms collaborate to pattern tissues. Although many details of the timing and coordination of patterning mechanisms remain to be determined, it is clear that during development cellular patterns arise from the integration of multiple patterning mechanisms, not from the exclusive use of one [1]. For example, in the development of the mammalian retina, axonal outgrowth, cell rearrangements, lateral inhibition and cell death all contribute to the creation of the regular pattern of retinal ganglion cells [4]. Similarly, in the development of the *Drosophila *eye, cell migration, lateral inhibition and multiple rounds of cell death must be coordinated to create the stunningly regular ommatidial pattern [5,6]. The development of serotonergic neurons in the ventral nerve cord of *Drosophila *requires the collaboration of cell selection, asymmetric division and apoptosis [7]. As a final example, cardiac development requires coordination of cell proliferation and apoptosis to create the embryonic outflow tract, cardiac valves, the conducting system and the coronary vasculature [8].

Some modeling studies have investigated the potential for multiple, coordinated patterning mechanism to create complex cellular patterns during development. In this work, a cellular pattern refers to the distribution of cell types in space. An early example of cellular pattern formation modeling is the work of Honda and Yamanaka [9] who examined the relationship between cellular growth and division in the formation of the polygonal cellular pattern of the avian oviduct epithelium. Another notable example is the work of Marée and Hogeweg [10] that investigated how individual cells of *Dictyostelium discoideum *organize to form the fruiting body. Their model beautifully simulated this complex morphogenetic process, and it required the joint operation of differential adhesion, cell differentiation, changes in cell rigidity, and the response of cells to a paracrine signaling molecule. The Maree-Hogeweg model provided the first clear insight into how the later stages of morphogenesis are achieved in this organism.

Eglen and Willshaw [4] examined the ability of lateral inhibition to create mosaic patterns of on- and off-center retinal ganglion cells that matched the regularity of biological mosaics in the cat retina. In contrast to many earlier studies, these investigators modeled arrays of irregularly-shaped cells rather than simulating cells as perfect hexagons. Beginning with an imperfect pattern of two cell types, they discovered that lateral inhibition alone was insufficient to create mosaics with the regularity seen in nature. They also found that cell death acting in isolation on the initial imperfect pre-pattern did not generate the regular pattern observed in the cat retina. Eglen and Willshaw hypothesized that lateral inhibition and cell death act sequentially to pattern the on- and off-center ganglion cells of the mammalian retina.

More recently, Izaguirre et al. [11] developed a multiple model software package for simulating morphogenesis. They termed this model *CompuCell *and used it in a pilot study to simulate vertebrate limb development. In this study, Izaguirre et al. [11] utilized modules that involve differential adhesion, reaction-diffusion, cell differentiation, and cell division. This work has recently been extended to understand chick wing development [12]. Taken together, these models demonstrate the necessity of multiple interacting mechanisms to accurately reproduce the development of complex components.

Finally, Salizar-Ciudad et al. [1][13] explored the development of mammalian teeth through a modified reaction-diffusion model. In this model, which considers epithelium and underlying mesenchyme, a diffusing activator and inhibitor create differentiated, non-growing enamel knot signaling centers in the epithelium. Epithelial cells and mesenchyme outside enamel knots grow in response to a signal originating from the knots. The unique feature of this model is that the growth of non-knot cells, which drives morphogenesis, alters the reach of the growth signal. In this way, the mechanisms of pattern formation (growth dependent on the concentration of the knot-centered signal) and morphogenesis are coupled in a dynamic feedback loop that produces the tooth.

**Figure 1:**
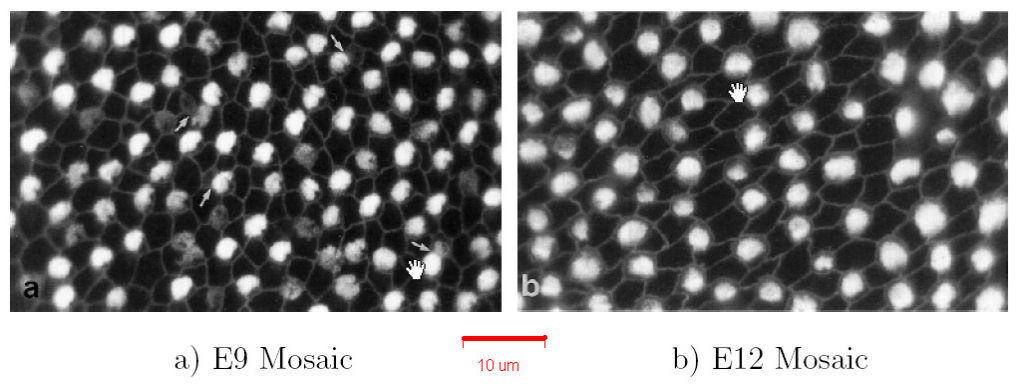
**Basilar papilla at E9 and E12**. Images of regular mosaics at embryonic day 9 (E9) and E12 (from [18]) in the basilar papilla. The spatial regularity of the primary cells (white) is significantly improved in E12, compared to E9.

We are interested in learning how regular mosaic patterns of two different cell types can form in epithelial sheets. These patterns are common in the embryo and are seen in such systems as the *Drosophila *neurectoderm [14,15] and eye [5], butterfly and moth wing scale cells and surrounding epithelial cells [16], insect sensory bristle cells and non-sensory epithelial cells [17], and sensory hairs and supporting cells of the vertebrate inner ear [18,19] (see Figure 1).

These mosaic patterns have been modeled [4,20-22], but often using one or at most two developmental patterning mechanisms. Previous studies have only partially explored the outcome of interactions between known patterning mechanisms or the possible outcome of feedback among mechanisms such as lateral inhibition, cell rearrangement driven by differential adhesion, and programmed cell death. In a recent review of developmental patterning, Salazar-Ciudad et al. [1] distinguish between morphostatic and morphodynamic strategies of patterning. In the morphostatic strategy, which is the basis of many existing models, an initial inductive mechanism is followed by a morphogenetic mechanism. Induction and morphogenesis operate independently and do not overlap in time. Induction involves intercellular signaling and morphogenetic mechanisms, as considered by Salazar-Ciudad et al. [1], include directed mitosis, differential growth, apoptosis, migration, and differential adhesion. In contrast, a morphodynamic strategy involves simultaneous operation of inductive and morophogenetic mechanisms to create pattern. One example of a morphodynamic mechanism is the combination of lateral inhibition, an inductive mechanism that involves signaling through membrane-bound molecules, with programmed cell death, a morphogenetic mechanism. In modeling this combination, lateral inhibition is used to establish cell fates and is followed by programmed cell death to refine a pattern of two cell types. This sequence is then repeated until a crisp pattern of cell types is achieved. In contrast to a morphostatic approach, as pattern emerges in a morphodynamic process, pattern elements acquire new signaling properties and in so doing influence the final form the pattern will take. The process is both iterative and dynamic.

In this work, we explore how the interplay between three widely-utilized patterning mechanisms – lateral inhibition, differential adhesion, and programmed cell death – can generate regular, mosaic patterns seen in development using biologically-realistic cells that dynamically change their shape and contact patterns. We find that combining all three processes into a network with feedback loops produces regular mosaics that are not achieved when lateral inhibition, differential adhesion, or programmed cell death operate independently or in simpler networks. Moreover, as these mechanisms are coupled, the robustness of pattern formation to alterations in cell-cell adhesive strength is increased. We compare the output of our models to the mosaic pattern of sensory and supporting cells of the developing chick basal papilla as reported by Goodyear and Richardson [18]. The power of this computational approach is that it allows exploration of the limits of individual pattern formation mechanisms and an examination of the potential offered by combining independent mechanisms in a variety of ways. This may inform thinking about the possible ways patterning mechanisms are deployed and coordinated to create mosaic patterns during development.

## Methods

### Implementation of the models

**Figure 2:**
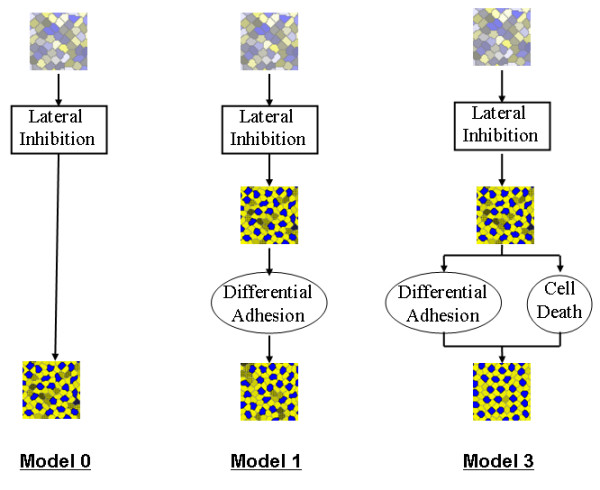
**Morphostatic computational models**. The three morphostatic computational models studied. Each model begins with the inductive mechanism of lateral inhibition run until a fixed point. Model 1 then runs differential adhesion. Model 3 follows lateral inhibition with the morphogenetic mechanisms of differential adhesion and cell death running together (interlaced in time). In the embryo, this is equivalent to the mechanisms running simultaneously.

**Figure 3:**
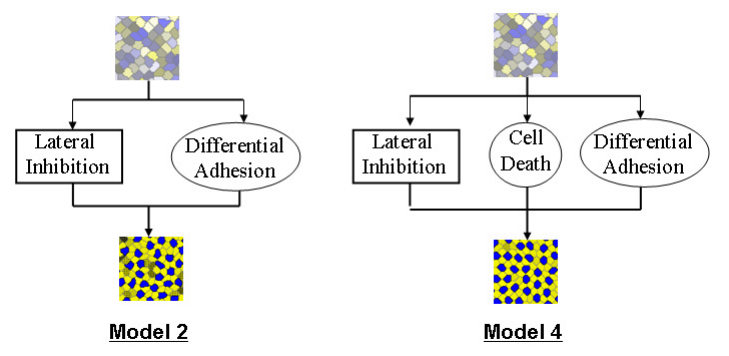
**Morphodynamic computational models**. The two morphodynamic computational models studied. Model 2 runs lateral inhibition and differential adhesion together (interlaced in time). Model 4 runs lateral inhibition, cell death and differential adhesion together. In the embryo, this is equivalent to the mechanisms running simultaneously.

The five models explored in this work are shown in Figure 2 and Figure 3. Each model employs one or more of three biologically-relevant pattern formation mechanisms: lateral inhibition, differential adhesion and programmed cell death. The input to each model is a 2D sheet of 100–400 irregularly-shaped cells expressing a random amount of each of two proteins (Notch and Delta) that mediate lateral inhibition.

**Model 0 **is a morphostatic model that executes lateral inhibition until a fixed point (no change in expression levels of Notch and Delta) and represents an extension of Collier et al. [22] to a natural arrangement of cells.

**Model 1 **is a morphostatic model that first uses lateral inhibition to determine cell fate, followed by cell rearrangement driven by differential adhesion.

**Model 2 **is a morphodynamic extension of Model 1, where lateral inhibition and differential adhesion form a feedback loop in which cell rearrangement and cell signalling are interlaced.

**Model 3 **is a morphostatic model that investigates the effect of lateral inhibition first determining cell fate, followed by a feedback loop of programmed cell death and rearrangement driven by differential adhesion.

**Model 4 **is a morphodynamic extension of Model 3, in which lateral inhibition is interlaced with programmed cell death and rearrangement.

Models were terminated at quiescence, with quiescence defined differently depending upon component mechanisms in each model. Models 1 and 2 were run until the cell defect rate (see below) showed no trend over 30 model iterations. Models 3 and 4 were run until no cell death occurred over 30 model iterations.

The implementation of each pattern forming mechanism and the method used to generate the random input patterns as the starting point of each model is described below.

#### Differential adhesion

Differential adhesion was simulated using the Cellular Potts Model (CPM) [21]. A principle advantage of this model is that global rearrangements within sheets of cells are emergent properties of local interactions between simple sub-cellular components. Each cell is represented as a set of contiguous lattice sites. Cell-cell contacts occur through adjacent lattice sites of different cells. In outline, the cells within the two-dimensional array have defined adhesive properties for each other and the surrounding medium. Cells may form new contacts and move with restrictions in size and in shape. All cell rearrangement is driven by a process of stochastic energy minimization.

The CPM is described by a Hamiltonian equation that estimates the total energy of a particular arrangement of cells. This equation is:

H=∑z¯,z¯′Jτσz¯,τσz¯′+∑σ(aσ−Aσ)2
 MathType@MTEF@5@5@+=feaafiart1ev1aaatCvAUfKttLearuWrP9MDH5MBPbIqV92AaeXatLxBI9gBaebbnrfifHhDYfgasaacPC6xNi=xI8qiVKYPFjYdHaVhbbf9v8qqaqFr0xc9vqFj0dXdbba91qpepeI8k8fiI+fsY=rqGqVepae9pg0db9vqaiVgFr0xfr=xfr=xc9adbaqaaeGacaGaaiaabeqaaeqabiWaaaGcbaGaemisaGKaeyypa0ZaaabeaeaacqWGkbGsdaWgaaWcbaacciGae8hXdq3aaSbaaWqaaiab=n8aZnaaBaaabaGafmOEaONbaebaaeqaaaqabaWccqGGSaalcqWFepaDdaWgaaadbaGae83Wdm3aaSbaaeaacuWG6bGEgaqegaqbaaqabaaabeaaaSqabaaabaGafmOEaONbaebacqGGSaalcuWG6bGEgaqegaqbaaqab0GaeyyeIuoakiabgUcaRmaaqababaGaeiikaGIaemyyae2aaSbaaSqaaiab=n8aZbqabaGccqGHsislcqWGbbqqdaWgaaWcbaGae83WdmhabeaakiabcMcaPmaaCaaaleqabaGaeGOmaidaaaqaaiab=n8aZbqab0GaeyyeIuoaaaa@4FC3@

The first term estimates the total surface energy between all contacting cells σz¯
 MathType@MTEF@5@5@+=feaafiart1ev1aaatCvAUfKttLearuWrP9MDH5MBPbIqV92AaeXatLxBI9gBaebbnrfifHhDYfgasaacPC6xNi=xH8viVGI8Gi=hEeeu0xXdbba9frFj0xb9qqpG0dXdb9aspeI8k8fiI+fsY=rqGqVepae9pg0db9vqaiVgFr0xfr=xfr=xc9adbaqaaeGacaGaaiaabeqaaeqabiWaaaGcbaacciGae83Wdm3aaSbaaSqaaiqbdQha6zaaraaabeaaaaa@2F5E@ and σz¯′
 MathType@MTEF@5@5@+=feaafiart1ev1aaatCvAUfKttLearuWrP9MDH5MBPbIqV92AaeXatLxBI9gBaebbnrfifHhDYfgasaacPC6xNi=xH8viVGI8Gi=hEeeu0xXdbba9frFj0xb9qqpG0dXdb9aspeI8k8fiI+fsY=rqGqVepae9pg0db9vqaiVgFr0xfr=xfr=xc9adbaqaaeGacaGaaiaabeqaaeqabiWaaaGcbaacciGae83Wdm3aaSbaaSqaaiqbdQha6zaaryaafaaabeaaaaa@2F69@ by summing Jτσz¯,τσz¯′
 MathType@MTEF@5@5@+=feaafiart1ev1aaatCvAUfKttLearuWrP9MDH5MBPbIqV92AaeXatLxBI9gBaebbnrfifHhDYfgasaacPC6xNi=xH8viVGI8Gi=hEeeu0xXdbba9frFj0xb9qqpG0dXdb9aspeI8k8fiI+fsY=rqGqVepae9pg0db9vqaiVgFr0xfr=xfr=xc9adbaqaaeGacaGaaiaabeqaaeqabiWaaaGcbaGaemOsaO0aaSbaaSqaaGGaciab=r8a0naaBaaameaacqWFdpWCdaWgaaqaaiqbdQha6zaaraaabeaaaeqaaSGaeiilaWIae8hXdq3aaSbaaWqaaiab=n8aZnaaBaaabaGafmOEaONbaeHbauaaaeqaaaqabaaaleqaaaaa@38EB@ over all adjacent lattice sites z¯
 MathType@MTEF@5@5@+=feaafiart1ev1aaatCvAUfKttLearuWrP9MDH5MBPbIqV92AaeXatLxBI9gBaebbnrfifHhDYfgasaacPC6xNi=xH8viVGI8Gi=hEeeu0xXdbba9frFj0xb9qqpG0dXdb9aspeI8k8fiI+fsY=rqGqVepae9pg0db9vqaiVgFr0xfr=xfr=xc9adbaqaaeGacaGaaiaabeqaaeqabiWaaaGcbaGafmOEaONbaebaaaa@2D68@ and z¯′
 MathType@MTEF@5@5@+=feaafiart1ev1aaatCvAUfKttLearuWrP9MDH5MBPbIqV92AaeXatLxBI9gBaebbnrfifHhDYfgasaacPC6xNi=xH8viVGI8Gi=hEeeu0xXdbba9frFj0xb9qqpG0dXdb9aspeI8k8fiI+fsY=rqGqVepae9pg0db9vqaiVgFr0xfr=xfr=xc9adbaqaaeGacaGaaiaabeqaaeqabiWaaaGcbaGafmOEaONbaeHbauaaaaa@2D73@ where σz¯≠σz¯′
 MathType@MTEF@5@5@+=feaafiart1ev1aaatCvAUfKttLearuWrP9MDH5MBPbIqV92AaeXatLxBI9gBaebbnrfifHhDYfgasaacPC6xNi=xH8viVGI8Gi=hEeeu0xXdbba9frFj0xb9qqpG0dXdb9aspeI8k8fiI+fsY=rqGqVepae9pg0db9vqaiVgFr0xfr=xfr=xc9adbaqaaeGacaGaaiaabeqaaeqabiWaaaGcbaacciGae83Wdm3aaSbaaSqaaiqbdQha6zaaraaabeaakiabgcMi5kab=n8aZnaaBaaaleaacuWG6bGEgaqegaqbaaqabaaaaa@34B9@; the second term implements an area constraint on cells where *a*_*σ *_is the actual area (the count of lattice sites, which may range between 64 and 144) of a cell *σ*, and *A*_*σ *_is *σ*'s target area. In these simulations, a lattice site represents approximately a 600 *nm *× 600 *nm *square, cells have diameters of approximately 8 *μm *and the total area of simulation is approximately 25, 600 *μ*^2^, based on dimensions given in [18].

Two cell types and the medium are considered in the CPM model implemented here. These are represented as *τ*_*σ *_= *p *for primary cells, *τ*_*σ *_= *s *for secondary cells, and *τ *= *m *for the medium. The area constraint is only applied to primary and secondary cells. A *J*_*τ*, *τ' *_matrix implements the relative surface tensions between the three types (primary cell, secondary cell, and medium), with J values inversely related to cell-cell or cell-medium adhesion. In experiments that examined the trajectory of mosaic pattern quality as each model ran, J values were fixed at: *J*_*p*, *p *_= 21, *J*_*s*, *s *_= 8, *J*_*s*, *p *_= 11, *J*_*p, m *_= 21 and *J*_*s, m *_= 21, similar to values used for the "checker board" mosaic rearrangement experiments reported by Graner and Glazier [21]. In experiments that investigated the robustness of the models under varying homotypic adhesive strengths, *J*_*s*, *s *_and *J*_*p*, *p *_were varied between 1 ≤ *J*_*s, s*_, *J*_*p*, *p *_≤ 21, *J*_*s*, *p *_= 11 and *J*_*s, m *_= *J*_*p, m *_= 21.

Low energy cell arrangements are determined by repeatedly copying the state of one lattice site to an adjacent lattice site for lattice sites belonging to different cells. Let Δ*H *be the change in energy resulting from the potential copy of one lattice site state. Then, if Δ*H *< 0, the state change is always accepted, and if Δ*H *= 0, the state change is accepted with probability 0.5. Otherwise the state change is accepted with probability e−ΔHT
 MathType@MTEF@5@5@+=feaafiart1ev1aaatCvAUfKttLearuWrP9MDH5MBPbIqV92AaeXatLxBI9gBaebbnrfifHhDYfgasaacPC6xNi=xH8viVGI8Gi=hEeeu0xXdbba9frFj0xb9qqpG0dXdb9aspeI8k8fiI+fsY=rqGqVepae9pg0db9vqaiVgFr0xfr=xfr=xc9adbaqaaeGacaGaaiaabeqaaeqabiWaaaGcbaGaemyzau2aaWbaaSqabeaacqGHsisldaWcaaqaaiabfs5aejabdIeaibqaaiabdsfaubaaaaaaaa@3200@, where *T *is the temperature, representing the agitation of the cells [21].

The CPM is used to create the random input pre-pattern for each of the 5 models and is then used repeatedly after lateral inhibition or programmed cell death in models 1–4 (see Figure 2 and Figure 3). The input pre-pattern is generated starting from a regular square grid of 20 × 20 cells, each composed of 12 × 12 lattice sites. The target area *A*_*σ *_of each cell is set to 144 ± *q*, where *q *is a normally distributed variable with a standard deviation of 12. The square grid is then annealed for 1000 Monte Carlo steps (MCS) at *T *= 10 (see [21] for more details), then 10 MCS at *T *= 0. The differential adhesion step in models 1–4 is implemented as 100 MCS at *T *= 5 followed by 10 MCS at *T *= 0.

### Lateral inhibition

**Figure 4:**
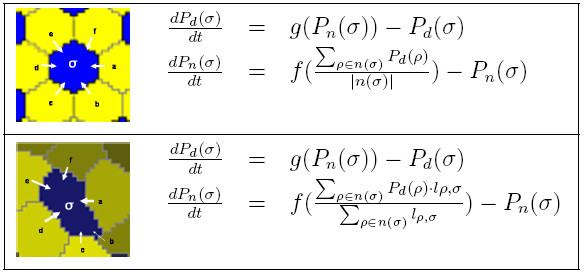
**Lateral inhibition model**. Comparison between the lateral inhibition models of Collier et al. [22] that employed hexagonal cells and the models used in this work with naturally shaped cells. In both models, *P*_*d*_(*σ*) (Delta) is driven to the opposite of *P*_*n*_(*σ*) (Notch) within each cell, while cell-cell communication across contacting membranes regulates *P*_*n*_(*σ*). The length of the common border between *σ *and *ρ *is *l*_*ρ*, *σ*_, which is the count of all 8-connected lattice sites between and *ρ*, and *σ*, g(x)=1(1+x2),f(x)=x2(1+x2)
 MathType@MTEF@5@5@+=feaafiart1ev1aaatCvAUfKttLearuWrP9MDH5MBPbIqV92AaeXatLxBI9gBaebbnrfifHhDYfgasaacPC6xNi=xH8viVGI8Gi=hEeeu0xXdbba9frFj0xb9qqpG0dXdb9aspeI8k8fiI+fsY=rqGqVepae9pg0db9vqaiVgFr0xfr=xfr=xc9adbaqaaeGacaGaaiaabeqaaeqabiWaaaGcbaGaem4zaCMaeiikaGIaemiEaGNaeiykaKIaeyypa0ZaaSaaaeaacqaIXaqmaeaacqGGOaakcqaIXaqmcqGHRaWkcqWG4baEdaahaaWcbeqaaiabikdaYaaakiabcMcaPaaacqGGSaalcqWGMbGzcqGGOaakcqWG4baEcqGGPaqkcqGH9aqpdaWcaaqaaiabdIha4naaCaaaleqabaGaeGOmaidaaaGcbaGaeiikaGIaeGymaeJaey4kaSIaemiEaG3aaWbaaSqabeaacqaIYaGmaaGccqGGPaqkaaaaaa@47BF@ and *n*(*σ*) returns the set of cells that are direct neighbors of *σ*.

Some early work implemented lateral inhibition using a strategy where a single randomly chosen cell is assigned a primary identity and its neighbors are assigned a secondary identity. This method is repeated until all cells are assigned [20]. Collier et al. [22] developed a more realistic model based on protein expression levels and cell-cell membrane signaling. They unitized perfectly hexagonal cells of fixed size. Our model extends this work to naturally shaped cells of varying size. For each cell, *σ*, let *P*_*d*_(*σ*) be the dimensionless expression of protein Delta, where 0 ≤ *P*_*d*_(*σ*) ≤ 1.0, and let *P*_*n*_(*σ*) be the dimensionless expression of Notch, where 0 ≤ *P*_*n*_(*σ*) ≤ 1.0. Initially all cell protein values are set from a uniform random distribution [0.5, 1.0]. This modeling of protein expression at the cell level (see Merks and Glazier [23]), rather than at the lattice site level, is appropriate since cell-cell signalling occurs only across contacting membranes. The interaction between adjacent cells is modeled as coupled differential equations shown in Figure 4.

The expression of *P*_*n *_implements cell-cell contact signalling, where each cell can sense the expression levels of *P*_*d *_of its immediate neighbors via their common membranes. In Collier et al. [22] cells were modeled as an exact hexagonal mesh, implying that the influence of each neighbor is equal. In naturally arranged cells, the influence of a neighbor cell *ρ *on the expression of *P*_*n*_(*σ*) is proportional to the length of the membrane shared between *σ *and *ρ*. A longer membrane means increased *P*_*n*_(*σ*) production as shown in the differential equations of Figure 4. The length of the common membrane between *σ *and *ρ*, *l*_*ρ*, *σ*_, is re-computed and cached following each cell rearrangement driven by a CPM-anneal.

**Figure 5:**
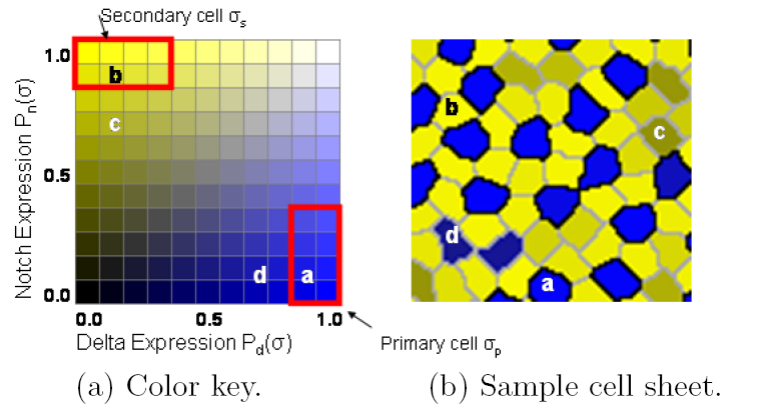
**Cell protein expression levels**. The color key used throughout the paper to denote the expression levels of Notch and Delta in each cell. Four cells labeled a, b, c and d are identified in the sheet of cells, and their corresponding expression levels shown in the color key. Cell a is a primary cell. Cell b is a secondary cell. Cell c is a defect since it is contacting only one primary cell. Cell d is a defect since it is touching another primary cell.

Lateral inhibition is run by numerically solving the differential equations using the Runge-Kutta method (with *dt *= 0.05) until a fixed point is reached where the average update error (the average difference in the protein values between iterations) is ≤ 10^-8 ^per cell. Once lateral inhibition is terminated, the type of each cell is determined by inspecting values of Notch and Delta as illustrated in Figure 5. A cell *σ *becomes secondary if *P*_*n*_(*σ*) ≥ 0.8 and *P*_*d*_(*σ*) ≤ 0.4. A cell becomes primary if *P*_*d*_(*σ*) ≥ 0.8 and *P*_*n*_(*σ*) ≤ 0.4. The default type for the cells is primary.

### Programmed cell death

**Figure 6:**
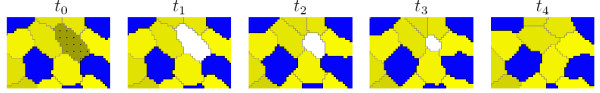
**Images showing cell death**. When the defect cell (checkered) dies, it becomes medium. As the remaining cells are annealed, cell adhesion causes the void to be filled, near-by cells to shift position and new cell-cell contacts are created and lengthened.

Programmed cell death occurs in Models 3 and 4 when cells autonomously determine that they are defective according to criteria discussed below. In cases where the mosaic contains two or more defect cells, only one of the cells is randomly selected to die at each iteration of the model. One cell is picked each model iteration to simplify the model and to avoid the need to introduce additional parameters. The space occupied by the dead cell is converted to medium and neighboring cells rearrange by differential cell adhesion to fill the space as illustrated in Figure 6.

Izaguirre et al. [11] modeled cell death by shrinking the target area of the dying cell. Potential complications of this method are the need to set a rate of target area reduction, and the fact that the shrinking cell maintains its original adhesive properties, thus drawing in surrounding cells. Modeling cell death by transforming the dead cell to medium may be a more realistic method of simulating death by apoptosis. Each iteration of cell death in the Models is followed by a fixed annealing period of 100 MCS. Models with cell death terminate after 30 iterations of differential adhesion (each 100 MCS) with no cell death.

### Evaluating the regularity of natural mosaics

Mosaic pattern development processes have evolved to produce a regular mosaic of primary cells that provide efficient sensory coverage for the eye [2,5,24], insect sensory bristles [17], vertebrate inner ear [18,19] and for structural uniformity, such as in the butterfly and moth wing scales [16]. In this study, mosaic regularity is evaluated based on two measures: the percentage of defect cells and the spatial regularity of the the primary cells.

#### Mosaic defects

Cell death is used in Models 3 and 4 to improve the spatial regularity of primary cells by selectively removing cells that disrupt the regular mosaic. Two principal questions are: (i) Which cells disrupt the spatial regularity constructed by lateral inhibition? and (ii) Is there a biologically feasible way in which such a defect cell could self-select and choose to die?

**Figure 7:**
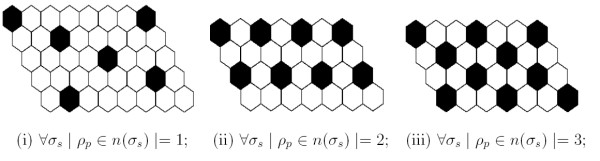
**Lateral inhibition solutions**. The three homogeneous states for the solution of the lateral inhibition model from [22]. Each solution is defined by the count of primary neighbors *ρ*_*p *_of each secondary cell *σ*_*s*_, where count is either 1, 2 or 3. Primary cells are black and secondary cells are white.

Ideally, developmental processes will produce a mosaic of regularly spaced primary cells, each surrounded by a single ring of secondaries. Such a regular array would be both efficient, in that the minimum number of primary cells are employed, and complete, in that the area of the mosaic would contain no gaps and be completely covered by sensory cells. Using an array of hexagonal cells, Collier et al. [22] analyzed the system of coupled differential equations implementing lateral inhibition and identified exactly three possible homogeneous solutions (reproduced in Figure 7), which we term solution type i, ii, or iii. If the mosaic consisted of a uniform population of only one of the solutions, a perfectly regular mosaic would result. However, due to random initial conditions and only local computation, the final mosaic consists of a mixture of all three solutions. This results in irregularities, even when modeling with uniform hexagonal cells. Moreover, with naturally shaped cells, an additional solution exists, in which two primary cells can touch when the shared membrane is short, termed solution type iv and illustrated in Figure 5(b).

With naturally shaped cells, lateral inhibition will produce a mosaic consisting of a randomly distributed mixture of all four possible solutions. In this work, we identify two solutions as disrupting the ideal pattern of a regular mosaic. First, solution type i where secondary cells only touch one primary (see Figure 7) will tend to push primary cells apart and create gaps, thereby reducing coverage. Second, solution type iv where primary-primary contacts will result in primary cells that are too close, thereby reducing efficiency. We propose that cell death and diffierential adhesion are utilized to eliminate cells from these two solution types, leaving a regular mosaic consisting of a mixture of only solution types ii and iii.

We define a defect cell as either a primary cell of solution type iv or a secondary cell of solution type i. This definition is supported by observations of biological mosaics, in particular by work of Goodyear and Richardson [18] (see Figure 1). Consider that at embryonic day 9, approximately 10% of secondary cells are of solution type i and 3% of primary cells were of solution type iv. In contrast, at embryonic day 12, no type i secondary cells or type iv primary cells were observed.

For the model to be biologically feasible, there must be a way for an individual cell to self-select as a defect and initiate programmed cell death. This determination can be made locally because a secondary cell which touches only one primary tends to express a non-saturated level of Notch (*P*_*n*_(*σ*) ≤ 0.8), while a secondary cell that touches two or three primary cells tends to express Notch at a saturated level (*P*_*n*_(*σ*) > 0.8). Such a defect cell is marked **c **in Figure 5. Similarly, due to mutual inhibition, a primary cell touching another primary will express a lower level of Delta compared with primary cells that contact only secondary cells. Such a cell is marked **d **in Figure 5. This local computation contrasts with the model of cell death described in [4] in which the decision to die was made globally, using criteria such as choosing the smallest or largest cell in the sheet. Significantly, Notch-mediated signaling is known to control apoptosis [25]. The model's use of low Notch levels to identify and trigger the death of defect cells is consistent with findings that inhibition or down-regulation of Notch induces apoptosis in murine erythroleukemia cells [26,27].

#### Measuring spatial regularity

Measures of spatial regularity include the regularity index [28] (sometimes referred to as the conformity ratio) and packing factor [29]. These measures were found by Eglen and Willshaw [4] to provide some discriminatory power in evaluating mosaics formed with and without cell death. However, the recent survey in da Fontoura Costa et al. [30] found that neither measure provided the needed sensitivity to discriminate between regular and irregular synthesized data and between center and peripheral agouti (*Dasyprocta agout*) retinal photoreceptor mosaics.

We evaluated the regularity index, packing factor and hexagonality index [30] to determine their sensitivity in discriminating between mosaics formed by all five models. We found that none of these measure is sufficiently sensitive to capture changes in regularity due to presence of defect cells. We developed a new regularity measure called the Voronoi Regularity Index (VRI) that exhibits high sensitivity in evaluating the mosaics produced by the models. To calculate VRI, a Voronoi tessellation is computed [31,32] over the center point (the centroid of the cell's lattice sites) of each primary cell. Let *D *be the set of distances between the center of each Voronoi cell and its vertices, then the VRI is the ratio of the mean of *D *divided by the standard deviation of *D*. VRI ranges from ∞ for perfect regularity to near 0 for no regularity.

## Results

We explored the effectiveness of the five models (Models 0 through 4 illustrated in Figure 2 and Figure 3) to create a regular two-dimensional mosaic pattern. In the first study, we compared the output of the models to the development of the mosaic of sensory (hair cells) and supporting cells of the chick basilar papilla reported by Goodyear and Richardson [18]. In the second study, we considered the robustness of the models under varying cell-cell adhesion values.

### Model performance simulating chick basilar papilla

The performance of each model was evaluated based on how well it simulated the mosaic of sensory and supporting cells of the chick basilar papilla. In this part of the study, the cell-cell and cell-medium adhesive strengths were fixed. We chose a set of *J *values similar to those used in Graner and Glazier [33]. These values result in negative surface tension between primary and secondary cells, and favor formation of mosaic patterns through differential adhesion. The values were *J*_*s*, *s *_= 8, *J*_*p, s *_= 11, *J*_*p*, *p *_= *J*_*p, m *_= *J*_*s, m *_= 21, giving surface tension values of *γ*_*p, s *_= -4.5, *γ*_*p, m *_= 17.0, *γ*_*s, m *_= 10.5 (calculation of surface tension from J values is given in [33]).

The baseline for model performance was the mosaic pattern created by one round of lateral inhibition (Model 0). The output of Model 0 is the input pattern for Models 1–4.

**Figure 8:**
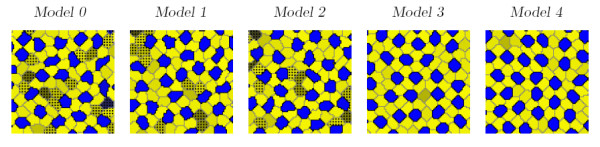
**Examples of model end state mosaics**. Examples of output mosaics formed by the 5 models. The cells are colored according to the key given in Figure 5. Defect cells are denoted by speckling.

**Figure 9:**
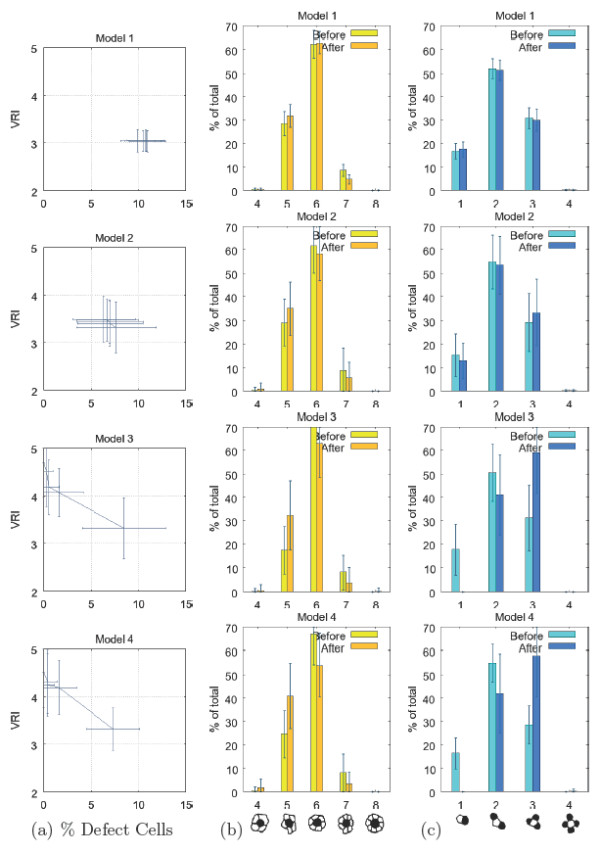
**Evaluation of mosaics**. Evaluation of input (before) and output (after) mosaics of 4 models. (a) Trajectories of defect rate (x axis) and VRI (y axis); (b) Change in the distribution of secondary cells around each primary; (c) Change in the distribution of primary cells around each secondary cell. Error bars are one standard deviation.

Five measures were made during and at the completion of each run of the models: the primary cell Voronoi regularity index (VRI), the number of secondary cells contacted by each primary cell, the number of primary cells contacted by each secondary cell, the ratio of secondary to primary cells, and the cell defect rate. Table 1 summarizes values of these measures and compares them with those measured in the chick basilar papilla by Goodyear and Richardson [18]. In the chick basilar papilla, shown in Figure 1, supporting cells correspond to secondary cells and sensory cells correspond to primary cells. Figure 8 shows example mosaics generated by the 5 models. Figure 9(a) shows the trajectory of VRI and defect rate during each model run, Figure 9(b) shows the distributions of the number of secondary cells around each primary cell and Figure 9(c) shows the number of primary cells around each secondary cell. Each model was run between 48 and 256 times. These results are considered below.

**Table 1:** Comparison of models

	CD E9	CD E12	Model 0	Model 1	Model 2	Model 3	Model 4
|*ρ*_*p *_∈ *n *(*σ*_*s*_)|	2.48 ± 0.07	3.07 ± 0.09	2.12 ± 0.69	2.16 ± 0.71	2.25 ± 0.66	2.46 ± 0.50	2.52 ± 0.50
|*ρ*_*s *_∈ *n *(*σ*_*p*_)|	4.56 ± 0.11	5.23 ± 0.16	5.81 ± 0.56	5.69 ± 0.51	5.65 ± 0.55	5.76 ± 0.56	5.68 ± 0.60
VRI	2.31	3.44	3.02 ± 0.24	3.08 ± 0.20	3.60 ± 0.54	4.50 ± 0.63	4.34 ± 0.61
|σs||σp| MathType@MTEF@5@5@+=feaafiart1ev1aaatCvAUfKttLearuWrP9MDH5MBPbIqV92AaeXatLxBI9gBaebbnrfifHhDYfgasaacPC6xNi=xH8viVGI8Gi=hEeeu0xXdbba9frFj0xb9qqpG0dXdb9aspeI8k8fiI+fsY=rqGqVepae9pg0db9vqaiVgFr0xfr=xfr=xc9adbaqaaeGacaGaaiaabeqaaeqabiWaaaGcbaWaaSaaaeaadaabdaqaaGGaciab=n8aZnaaBaaaleaacqWGZbWCaeqaaaGccaGLhWUaayjcSdaabaWaaqWaaeaacqWFdpWCdaWgaaWcbaGaemiCaahabeaaaOGaay5bSlaawIa7aaaaaaa@38F3@	1.85 ± 0.05	1.71 ± 0.05	2.83 ± 0.10	2.81 ± 0.11	3.00 ± 0.05	2.23 ± 0.07	2.23 ± 0.075
Defect Rate	9.00 ± 1.00	0.00 ± 0.00	8.69 ± 3.09	9.55 ± 0.64	5.81 ± 2.99	0.00 ± 0.00	0.00 ± 0.00

Comparison of the central distal (CD) chick basilar papilla cell mosaic development studied in [18] with model results. |*ρ*_*p *_∈ *n*(*σ*_*s*_)| is the average count of primary cells around each secondary cell, |*ρ*_*s *_∈ *n*(*σ*_*p*_)| is the average count of secondary cells around each primary, and |σs||σp|
 MathType@MTEF@5@5@+=feaafiart1ev1aaatCvAUfKttLearuWrP9MDH5MBPbIqV92AaeXatLxBI9gBaebbnrfifHhDYfgasaacPC6xNi=xH8viVGI8Gi=hEeeu0xXdbba9frFj0xb9qqpG0dXdb9aspeI8k8fiI+fsY=rqGqVepae9pg0db9vqaiVgFr0xfr=xfr=xc9adbaqaaeGacaGaaiaabeqaaeqabiWaaaGcbaWaaSaaaeaadaabdaqaaGGaciab=n8aZnaaBaaaleaacqWGZbWCaeqaaaGccaGLhWUaayjcSdaabaWaaqWaaeaacqWFdpWCdaWgaaWcbaGaemiCaahabeaaaOGaay5bSlaawIa7aaaaaaa@38F3@ is the ratio of the number of secondary cells by the number of primary cells. Error bands are one standard deviation, based on 40 random repeats of each model.

#### Trajectory of models

Model 1, which uses multiple rounds of differential adhesion to drive cell rearrangements, yielded no improvement in primary cell mosaic regularity (VRI) and a slight increase in defect rate during the model run. Model 2 showed a slight improvement in defect rate. In contrast, Models 3 and 4, which utilize death to eliminate defect cells, showed a clear trend in the improvement of VRI as defect cells die. There was a high degree of variation in both cell defect rate and VRI in runs of all four models. The trend for improvement in both measures was clear in Models 3 and 4, and though both model outputs display a high degree of variability, the improvement in cell defect rate and VRI for these two models was statistically significant based on a standard two-tailed t-test, with *p *< 0.05.

We also analyzed the VRI and defect rate in the published images of Goodyear and Richardson [18] that show primary and secondary cells of the central distal region of the chick basilar papilla between embryonic day 9 (E9) and day 12 (E12) (see Figure 1). The mosaic of hair and supporting cells emerges and is refined during this period of development. Between E9 and E12, the cell defect rate decreases from 9.00 ± 1.00 to 0.00 ± 0.00 and the VRI increases from 2.31 to 3.44 (Table 1). If E9 is considered to be the equivalent of the starting point of the models (i.e., Model 0), then the output patterns of Models 1 and 2, which contain residual defect cells, do not effectively simulate basilar papilla pattern development. This implies that lateral inhibition and differential adhesion are insufficient to explain the refinement of the primary cell mosaic in the chick basilar papilla observed by Goodyear and Richardson [18].

The VRI of primary cell mosaics generated by all the models is higher than that observed for basilar papilla at E9 (see Table 1). There is a modest increase in VRI in Models 1 and 2 (1.02- and 1.19-fold, respectively). There is an identical 1.49-fold increase in the VRI between E9 and E12 in the chick basilar papilla and in Model 3. The increase in VRI achieved in Model 4 is very similar (1.44-fold).

#### Cell contact patterns

In the hair cell/supporting cell mosaic of chick basilar papilla and in the four experimental models tested here, there is a trend toward an increased number of primary cells that are contacted by each secondary cell (Figure 9(c) and Table 1 row |*ρ*_*p *_∈ *n*(*σ*_*s*_)|), especially in Models 3 and 4. Goodyear and Richardson [18] observed a statistically significant increase in number of primary cells surrounding each secondary cell (|*ρ*_*p *_∈ *n*(*σ*_*s*_)|) from 2.48 ± 0.07 to 3.07 ± 0.09 in the central distal region of the papilla between E9 and E12. Of particular interest is the elimination by E12 of contacts between secondary cells and only one primary cell. The same result is achieved in Models 3 and 4 through cell death.

Values of the related measure of the average number of secondary cells contacted by each primary cell (the mean of |*ρ*_*s *_∈ *n*(*σ*_*p*_)|) were similar in the basilar papilla and in the model output. The significant increase in the number of contacts observed between E9 and E12 in the chick was not observed as models became more complex. In fact, the highest mean of |*ρ*_*s *_∈ *n*(*σ*_*p*_)| was observed for Model 0.

**Figure 10:**
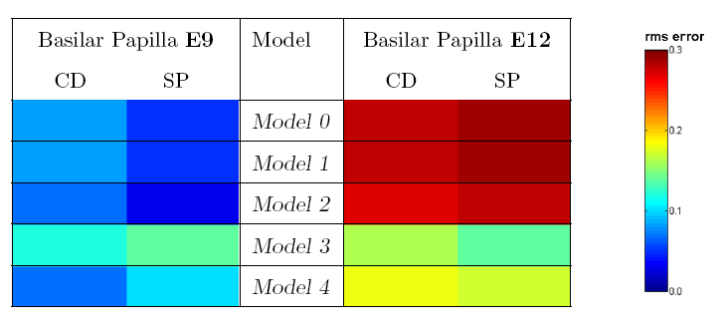
**Comparison between cell connectivity patterns generated by the models and observed in the chick basilar papilla**. The horizontal axis represents the alternatives studied in [18]. On the left are the observations from embryonic day E9 and the right observations from embryonic day E12. Measurements from two distinct spatial regions CD (central distal) and SP (superior proximal) are given. The vertical axis represents the end-states of Models 0 through 4. To compare each basilar papilla region with each model output, the measurements of secondary-primary cell connectivity are matched by computing the root mean squared error (rms). This rms error is calculated by comparing the distribution of the number of primary cells around each secondary cell. Data for the basilar papilla is taken from the graphs shown in the right column of Figure 11 from [18]. Data for the models is taken from the graphs given in Figure 9(c).

A match of cell contact distributions between the 5 model outputs and the centeral distal (CD) and superior proximal (SP) regions of the basilar papilla reported in [18] was performed by computing the average root mean squared error between each pair of distributions. The results are shown in Figure 10, which compares the distributions of primary cells around each secondary cell (no significant differences were found when comparing the distribution of secondary cells around each primary cell). At E9, models without cell death (Models 0, 1, and 2) best match observations reported in [18]. Only models with cell death (Models 3 and 4) have a close match to the E12 pattern, implying that cell death is necessary for the formation of the mosaic pattern found in the CD and the SP regions.

### Robustness of models

In this part of the study, we examined the robustness of Models 1 – 4 across a range of cell-cell adhesive values. While the regulation of cell-cell adhesive values is known to be a key element of morphogenesis in many systems, it is unclear how precisely these values must be specified to permit the emergence of the required pattern. We examined this issue in the second part of the empirical study.

**Figure 11:**
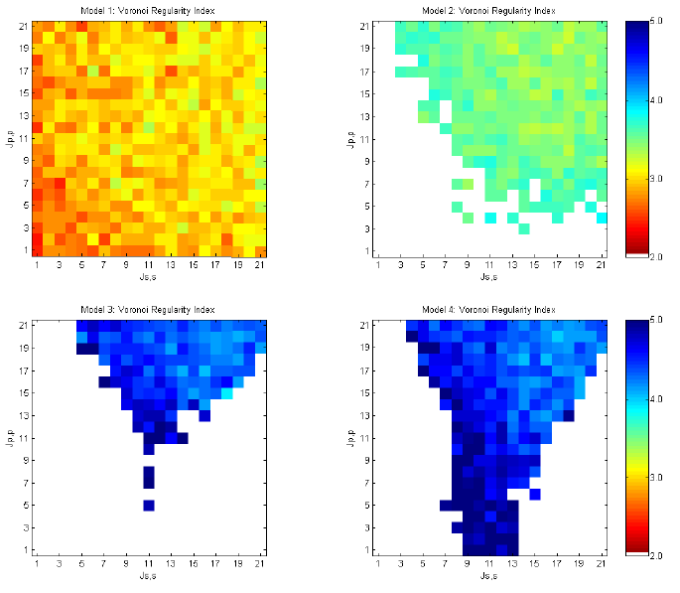
**Voronoi regularity index for models 1 – 4 run over varying cell-cell adhesive values**. Mosaics with *V RI *≥ 3.4 represent regularities found in chick basilar papilla at E12 from [18]. White areas represent unstable regions that do not produce solutions. Each graph cell shows the average of 40 randomized runs.

Values of primary cell-primary cell (*J*_*p, p*_) and secondary cell-secondary cell (*J*_*s, s*_) adhesiveness were varied across all pairs of integer values 1 ≤ *J*_*p, p*_, *J*_*s, s*_, ≤ 21. In every case, the primary cell-secondary cell adhesive value, *J*_*p*, *s *_, was fixed at 11 and cell-medium adhesiveness, *J*_*p, m *_and *J*_*s, m*_, was fixed at 21. We expect that robustness is a mark of a superior system for pattern formation. We observed strong differences in the robustness of the models to changes in cell adhesive strength. These results are shown in Figures 8, 9, 10, 11.

Four measures were used to compare the performance of the different models in producing regular mosaics. Only one, primary cell VRI, could be used to compare all the models. All other measures (cell defect rate, cell state change, and death rate) allowed only pair-wise comparisons between models. For example, because Models 3 and 4 run until no cell defects remain, the measure of cell defect rate is meaningless for these models. Similarly, cell death rate is only a valid measure for Models 3 and 4, the only ones that implement cell death. Even with a restriction to pair-wise comparisons, significant conclusions can be drawn about the robustness of all four models.

#### Primary cell Voronoi regularity index

As shown in Figure 11, the four models performed very differently regarding the range of adhesive values that allow production of a regular pattern of primary (sensory) cells. The morphostatic Model 1, with a single round of lateral inhibition followed by multiple rounds of cell rearrangements, did not produce a regular mosaic relative to more complex models. The morphodynamic Model 2, where lateral inhibition and differential adhesion form a feedback loop, produced a more regular mosaic over a range of adhesive values that span roughly the upper right quadrant of the graph (i.e., approximately *J*_*p*, *p *_and *J*_*s*, *s *_≥ 9). These correspond to negative *γ*_*p, s *_(see [33]) and homotypic cell-cell adhesive strengths that are lower than the fixed heterotypic affinity of primary and secondary cells (*J*_*p, s *_= 11). Outside this range of adhesive values, Model 2 became unstable with feedback between lateral inhibition and differential adhesion producing continuous changes between primary and secondary cell state (shown as white in Figure 11 and discussed below).

Model 3 is a morphostatic model that utilizes one round of lateral inhibition followed by an iterative loop of programmed cell death and cell rearrangements driven by differential cell adhesion. It produced a significantly more regular mosaic than Model 2. However, Model 3 failed across significant regions of cell-cell adhesive strength due to excessive cell death. This caused the initial sheet of 400 cells to be reduced to less than 10 cells. We term this a death cascade and it occurs at values of *J*_*s*, *s *_≲ 7 and *J*_*p*, *p *_≤ *J*_*p*, *s *_.

Model 4 is morphodynamic model where lateral inhibition forms a feedback loop with programmed cell death and cell rearrangements driven by differential cell adhesion. It produced equally regular mosaics as Model 3, but it expanded the range of usable adhesive values into regions where *J*_*p*, *p *_≤ *J*_*p*, *s *_.

#### Cell defect rate

**Figure 12:**
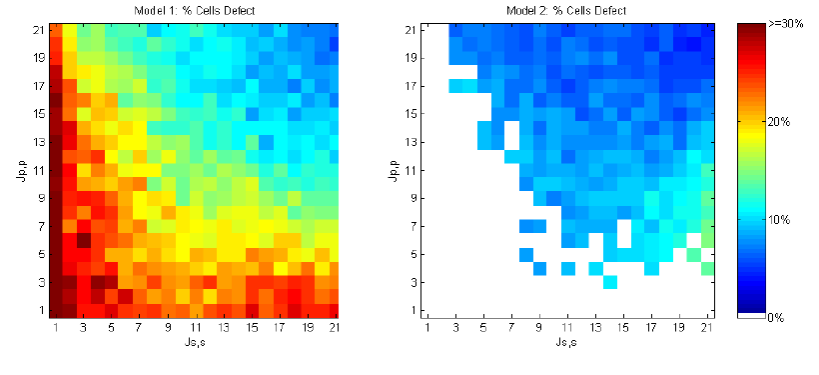
**Cell defect rates for models 1 and 2 run over varying cell-cell adhesive values**. Each graph cell shows the average of 40 randomized runs. White areas for Model 2 represent unstable regions that do not produce solutions.

Models 1 and 2 are run until the defect rate stabilizes, defined when the percentage of defect cells shows no trend over 30 model iterations. Figure 12 shows the defect rate at termination of Models 1 and 2 as a function of cell adhesive strength. By comparison, the default defect rate of Model 0 is ≈ 10%, which forms the input mosaic of Model 1 and Model 2.

Model 1 has a limited range of adhesive values for which the cell defect rate improves. This range roughly spans *γ*_*p, s *_≤ -3 (when Js,s+Jp,p2
 MathType@MTEF@5@5@+=feaafiart1ev1aaatCvAUfKttLearuWrP9MDH5MBPbIqV92AaeXatLxBI9gBaebbnrfifHhDYfgasaacPC6xNi=xH8viVGI8Gi=hEeeu0xXdbba9frFj0xb9qqpG0dXdb9aspeI8k8fiI+fsY=rqGqVepae9pg0db9vqaiVgFr0xfr=xfr=xc9adbaqaaeGacaGaaiaabeqaaeqabiWaaaGcbaWaaSaaaeaacqWGkbGsdaWgaaWcbaGaem4CamNaeiilaWIaem4CamhabeaakiabgUcaRiabdQeaknaaBaaaleaacqWGWbaCcqGGSaalcqWGWbaCaeqaaaGcbaGaeGOmaidaaaaa@37CD@ ≥ 14). and *J*_*p*, *p *_≥ *J*_*s, p*_. In contrast, Model 2 produced mosaics with net improvement of the default rate over a much broader range, roughly *γ*_*p, s *_≤ 0 (when Js,s+Jp,p2
 MathType@MTEF@5@5@+=feaafiart1ev1aaatCvAUfKttLearuWrP9MDH5MBPbIqV92AaeXatLxBI9gBaebbnrfifHhDYfgasaacPC6xNi=xH8viVGI8Gi=hEeeu0xXdbba9frFj0xb9qqpG0dXdb9aspeI8k8fiI+fsY=rqGqVepae9pg0db9vqaiVgFr0xfr=xfr=xc9adbaqaaeGacaGaaiaabeqaaeqabiWaaaGcbaWaaSaaaeaacqWGkbGsdaWgaaWcbaGaem4CamNaeiilaWIaem4CamhabeaakiabgUcaRiabdQeaknaaBaaaleaacqWGWbaCcqGGSaalcqWGWbaCaeqaaaGcbaGaeGOmaidaaaaa@37CD@ ≥ 11) and *J*_*p*, *p *_≥ 5 or about 70% of the range of homotypic cell adhesive strengths. These results demonstrate the ability of morphodynamic systems to extend the quality and robustness achieved by morphostatic systems that utilize the same developmental mechanisms. Significantly, the unstable region of homotypic cell adhesive strengths in Model 2 corresponds to the region of high defect rate (≥ 20%) in Model 1. Because Models 3 and 4 are run until there are no defects, they are not included in this comparison.

#### Instability of cell state

**Figure 13:**
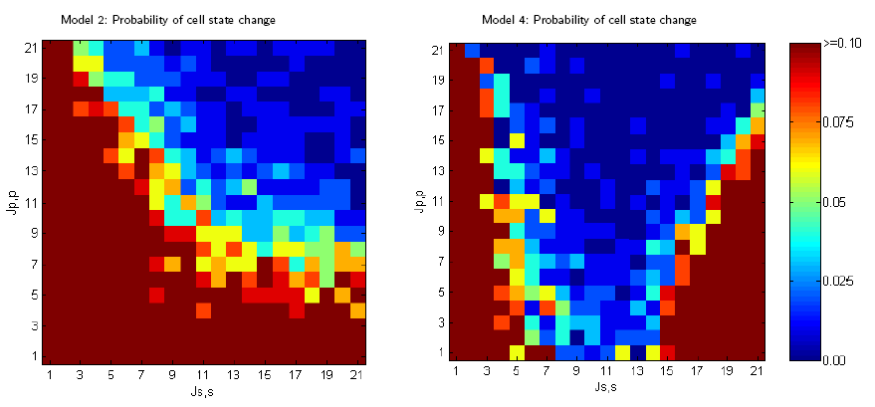
**Cell state stability for models 2 and 4 run over varying cell-cell adhesive values**. Cell state stability of Model 2 and Model 4. Each graph cell shows the average of 40 randomized runs. The probability of a cell changing its state as a function of homotypic adhesive strength is calculated by examining the last 30 model iterations and determining the proportion that involved any cell state change. The last 30 model iterations correspond in Model 2 to a period when the defect rate is stable and in Model 4 to a period when cell rearrangement is producing no defect cells and therefore no cell death.

Models 2 and 4 revealed an emergent property with potential biological significance: instability of cell state, where cells continually switch between primary and secondary states (See Figure 13). Although the endpoints of these two models differed (no trend in defect rate for Model 2 and no cell defects for Model 4), both exhibited instability in cell state determination at particular homotypic cell adhesive strengths. This was evident in dramatic reversals of primary and secondary cell identity. Such instability is shown in the video [34] that documents a run of Model 2. Models 2 and 4 are morphodynamic and incorporate a feedback loop between lateral inhibition and cell rearrangement. As a consequence of allowing a new round of lateral inhibition, many cells change their identity from primary to secondary and vice versa, based on the new cell contacts established by differential adhesion.

#### Programmed cell death

**Figure 14:**
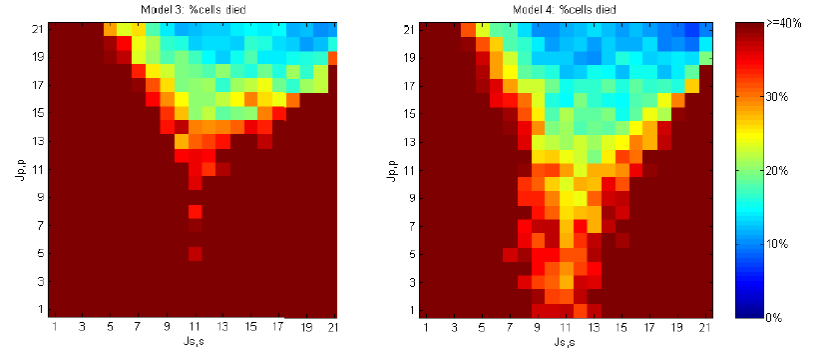
**Cell death rates for models 3 and 4 run over varying cell-cell adhesive values**. The death rates needed to achieve stable 0% defect rate. Adhesive values vary 1 ≤ *J*_*p*, *p *_≤ 22 and 1 ≤ *J*_*s*, *s*_, ≤ 22, with *J*_*s*, *p*_, = 11. Note that the red regions are where a death cascade occurs. Each graph cell shows the average over 40 randomized runs.

Figure 14 shows the percentage of cells that die as a function homotypic adhesion in Models 3 and 4. Both models are terminated when 30 iterations result in no programmed cell death and the resulting mosaic is free of any defect cells. For much of the homotypic adhesion space, high levels of cell death are required to reach termination. In substantial regions of the adhesion space almost all cells die. Such a death cascade is shown in the video in [34]. Death cascades occur when cell rearrangements after a cell death tend to create more defects. Although relatively high levels of programmed cell death (e.g., perhaps as high as 50% in the developing mammalian retina [5,6]) are observed, a system in which nearly all cells die is unlikely to be effective in pattern generation.

Figure 14 demonstrates that the morphodynamic Model 4 is more robust to differences in homotypic cell adhesive strength than the morphostatic Model 3. When lateral inhibition is incorporated into a loop with programmed cell death, subsequent defects caused by cell rearrangement can often be repaired by signalling over the new cell-cell contacts, thereby avoiding additional cell death. This process enables Model 4 to terminate successfully in regions of adhesive affinity where *J*_*s*, *s *_≈ *J*_*p*, *s*_, which result in death cascades in Model 3.

## Discussion

What mechanisms are needed to create a regular two-dimensional mosaic of cells? We explored answers to this question by examining the interplay of lateral inhibition, cell rearrangements driven by differential adhesion, and programmed cell death in creating and patterning two cell types into a regular mosaic. The performance of four experimental models that weave these patterning mechanisms together in different ways was assessed and compared with the development of a biological mosaic pattern, the regular array of sensory and supporting cells that emerges in the embryonic chick basilar papilla.

In the first part of this study, the output of four experimental models was compared with two mosaic patterns: the pattern generated computationally by a single round of lateral inhibition and the mosaic of sensory and supporting cells that emerges and is refined between embryonic days 9 and 12 in the chick basilar papilla. Modeling studies were performed at fixed values of adhesive strength chosen through guidance from the literature [33].

In early work modeling lateral inhibition, Collier et al. [22] showed that beginning from a field of equipotent hexagonal cells, lateral inhibition alone was insufficient to generate a perfectly regular mosaic of primary and secondary cells. They demonstrated that irregularity in the resulting mosaic was due to the random mixing of three alternative patterns corresponding to alternative homogeneous steady-state solutions to the differential equations.

In contrast to the geometrically perfect hexagonal cells of Collier et al. [22], our models use the Cellular Potts Model (CPM) to generate a population of irregularly-shaped, undifferentiated cells. A refinement of our approach is that the strength of signaling between a cell and each of its neighbors is weighed by the length of contacting membranes. Working with this more realistic model, we confirmed the results of Eglen and Willshaw [4] and showed that lateral inhibition working in isolation can create an irregular mosaic like those seen at early stages of development.

Using the VRI as a measure of the primary cell mosaic regularity, the average VRI of the mosaics created by lateral inhibition alone (Model 0) was 3.02, slightly less than the primary cell VRI of 3.44 observed in the E12 chick basilar papilla. Allowing iterations of lateral inhibition and cell rearrangements driven by differential adhesion (Model 2) improved primary cell regularity to 3.60. Incorporating the programmed death of cells that make defective contacts (Models 3 and 4), further improved primary cell regularity to values (4.50 and 4.35, respectively) above that observed in the chick basilar papilla.

In their description of the development of the chick basilar papilla sensory and supporting cell mosaic, Goodyear and Richardson [18] argued that cell rearrangements must occur after an initial pattern is created by lateral inhibition. Our results support this conclusion, but suggest that lateral inhibition and differential adhesion are insufficient to explain the connectivity distribution (where no secondary cells touch a single primary) seen in the E12 stage of the basilar papilla [18]. Goodyear and Richardson [18] used a rough numerical argument based on a field of 1000 cells to discount cell death as a mechanism in the development of the basilar papilla, but they assumed that cell death was limited to the elimination of one member of a pair of contacting primary cells. In contrast, in this work cell death occurs principally in secondary cells and accounts for the observed elimination of those secondary cells that contact exactly one primary as observed in [18]. Additionally, death of secondary cells would have the effect of decreasing the secondary to primary cell ratio as the pattern refines between E9 and E12. This is exactly what Goodyear and Richardson reported. Our results imply that cell death is necessary to account for the mosaic regularity and connectivity pattern observed in the basilar papilla.

In the second part of this study, we examined the robustness of each model to alterations in homotypic cell affinity. Others have explored how cell rearrangements are affected by varying levels of affinity between two different cell types and the surrounding medium [21,35]. However, to our knowledge, this study is the first to investigate how additional patterning mechanisms working in conjunction with differential cell adhesion perform over a range of adhesive values.

Mosaic formation by differential adhesion is favored when the average affinity between primary cells is less than the average affinity of primary-primary and secondary-secondary interactions, which in turn is less than the strength of primary-secondary interactions (i.e., (*J*_*p*, *p *_> (Js,s+Jp,p)2
 MathType@MTEF@5@5@+=feaafiart1ev1aaatCvAUfKttLearuWrP9MDH5MBPbIqV92AaeXatLxBI9gBaebbnrfifHhDYfgasaacPC6xNi=xH8viVGI8Gi=hEeeu0xXdbba9frFj0xb9qqpG0dXdb9aspeI8k8fiI+fsY=rqGqVepae9pg0db9vqaiVgFr0xfr=xfr=xc9adbaqaaeGacaGaaiaabeqaaeqabiWaaaGcbaWaaSaaaeaacqGGOaakcqWGkbGsdaWgaaWcbaGaem4CamNaeiilaWIaem4CamhabeaakiabgUcaRiabdQeaknaaBaaaleaacqWGWbaCcqGGSaalcqWGWbaCaeqaaOGaeiykaKcabaGaeGOmaidaaaaa@397F@ > *J*_*s, p*_). In contrast, when *J*_*p*, *p *_<(Js,s+Jp,p)2
 MathType@MTEF@5@5@+=feaafiart1ev1aaatCvAUfKttLearuWrP9MDH5MBPbIqV92AaeXatLxBI9gBaebbnrfifHhDYfgasaacPC6xNi=xH8viVGI8Gi=hEeeu0xXdbba9frFj0xb9qqpG0dXdb9aspeI8k8fiI+fsY=rqGqVepae9pg0db9vqaiVgFr0xfr=xfr=xc9adbaqaaeGacaGaaiaabeqaaeqabiWaaaGcbaWaaSaaaeaacqGGOaakcqWGkbGsdaWgaaWcbaGaem4CamNaeiilaWIaem4CamhabeaakiabgUcaRiabdQeaknaaBaaaleaacqWGWbaCcqGGSaalcqWGWbaCaeqaaOGaeiykaKcabaGaeGOmaidaaaaa@397F@ <*J*_*s, p*_, like cells tend to aggregate. The ability of each model to generate mosaic patterns, especially in the range of adhesive values unfavorable to mosaic formation, provided an assessment of the robustness of each patterning strategy. We found that the set of models performed quite differently across the examined range of homotypic cell adhesive values.

The primary cell VRI was examined for all four experimental models, and measures of the percentage of defective cell contacts, instability of cell state, and percentage of cell death required before model termination were examined in select pairs of models. Model 1, which ran only rounds of differential cell adhesion after one round of lateral inhibition, was the only one that provided stable solutions over the entire range of homotypic adhesive values.

Morphostatic Model 3, which ran a loop composed of cell death and differential adhesion without lateral inhibition, was notably sensitive to adhesive values. While each cell death event reduced the count of defect cells by one, subsequent cell rearrangements often caused additional defects, thereby creating a death cascade that terminated when only a few cells remained.

Morphodynamic Models 2 and 4, which incorporate differential adhesion and lateral inhibition into iterative loops, also failed at unfavorable homotypic adhesive values because of cell state instability. In these models, lateral inhibition repaired defective cell contacts formed by differential adhesion (e.g., two primary cells touching or a secondary cell not touching a primary cell). Iterating each model leads to an unstable cycle of mosaic disruption and repair.

Of the four experimental models, Model 4, which iterates a loop of lateral inhibition, programmed cell death, and differential adhesion, provided the highest mosaic regularity over the broadest range of homotypic adhesive values. The combination of programmed cell death and lateral inhibition was able to correct many pattern defects introduced by differential adhesion run at non-optimal values.

## Conclusion

We found that lateral inhibition acting alone (Model 0) was insufficient to create a highly regular mosaic of cells of biologically-realistic shape. Adding cell rearrangement driven by differential adhesion (Model 1) to the patterning strategy improved mosaic regularity. Strategies that included programmed cell death (Models 3 and 4) performed even better, yielding mosaic patterns more regular than those seen in the chick basilar papilla. Morphodynamic models that incorporated lateral inhibition into a feedback loop were unstable in some regions (Model 2), but when all three patterning mechanisms were utilized (Model 4), patterns became significantly more robust over a variety of homotypic cell-cell adhesion strengths. Finally, regular patterns could be generated using local computation based on units of single cells (as discussed by Merks and Glazier [23]); there was no need, as was the case in some earlier models [4], for global decisions such as choosing the nearest neighbor among a group of contacting cells or distinguishing the largest or smallest cell in a developmental field.

Individually, each of the patterning mechanisms examined here has well documented roles in development. What is less clear is the temporal overlap and coordination between these mechanisms during development. In exploring how lateral inhibition, cell rearrangements driven by differential cell adhesion, and programmed cell death can work alone or together, we found that when using cells of irregular size and shape, lateral inhibition is insufficient to create mosaic patterns with the regularity seen in nature. Coupling differential adhesion with lateral inhibition in an iterative loop raised mosaic regularity to the level observed in our target pattern, the mosaic of sensory and supporting cells of the chick basilar papilla. Further improvement in regularity was achieved when programmed cell death was added to the models. These results support the view that patterning mechanisms are used together in a temporally-overlapping and iterative manner in biological development.

The specification of characteristic cell adhesive values through the expression of cell surface adhesive proteins is a well established element of patterning. In examining how homotypic cell adhesion affects the patterns generated by the various models, we found that a strategy that incorporates programmed cell death and lateral inhibition into a loop with differential adhesion (Model 4) is robust over a broad range of adhesive values, including some values that would not allow development of a regular mosaic under simpler schemes (Models 1, 2 and 3). This finding suggests that in addition to improving mosaic regularity, patterning strategies that incorporate multiple interacting mechanisms offer the advantage of robustness in the face of poorly specified or highly variable initial conditions.
